# Facile preparation of a tetraethylenepentamine-functionalized nano magnetic composite material and its adsorption mechanism to anions: competition or cooperation†

**DOI:** 10.1039/c8ra00237a

**Published:** 2018-03-16

**Authors:** Meiqin Hu, Haoyu Shen, Si Ye, Yan Wang, Jiali Zhang, Shanshan Lv

**Affiliations:** Ningbo Institute of Technology, Zhejiang University Ningbo Zhejiang 315100 China hyshen@nit.zju.edu.cn

## Abstract

A tetraethylenepentamine (TEPA)-functionalized nano-Fe_3_O_4_ magnetic composite material (nFe_3_O_4_@TEPA) was synthesized by a facile one-pot solvothermal method. It was characterized by elementary analysis (EA), powder X-ray diffraction (XRD), Fourier transform infrared spectrometry (FTIR), transmission electron microscopy (TEM) and vibrating sample magnetometry (VSM). The results show that the nFe_3_O_4_@TEPA has an average size of ∼20 nm, with a saturation magnetization intensity of 48.2 emu g^−1^. Its adsorption properties were investigated by adsorbing fluorine ions, phosphate, Cr(vi) and their co-existing water system. The adsorption performance was studied as a function of solution pH, initial concentration of ions, contact time and temperature for each ion. The adsorption of the multi-ion co-existing system was studied *via* batch tests, XPS and FTIR analyses. The effect of co-existing ions was studied through Box-Behnken Design (BBD) and response surface methodology (RSM). It can be deducted that the adsorption mechanism of an individual fluorine ion or phosphate was mainly related to electrostatic attraction, while that of Cr(vi) might be mainly related to electrostatic attraction and coordination interactions. For the fluorine ion and phosphate bi-component system, their adsorption was competitive *via* ion exchange. For the Cr(vi), fluorine ion and phosphate tri-component co-existing system, Cr(vi) took priority for adsorption and could replace the absorbed fluorine ion or phosphate by competitive reaction, but not *vice versa*.

## Abstract 2

A tetraethylenepentamine (TEPA)-functionalized nano-Fe_3_O_4_ magnetic composite material (nFe_3_O_4_@TEPA) was synthesized by a facile one-pot solvothermal method.

## Graphical abstract

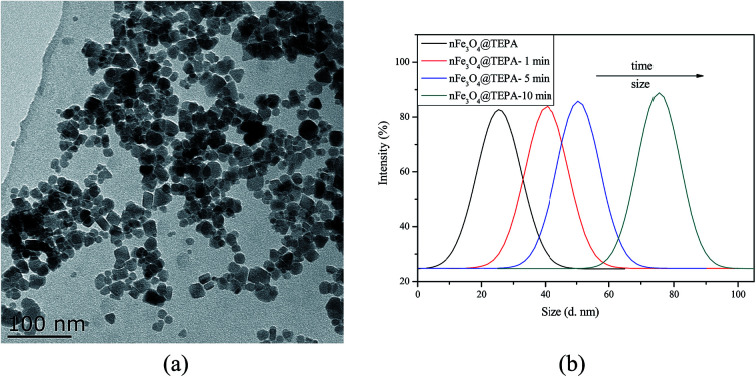

## 1 Introduction

Elevated levels of oxyanions (*e.g.* arsenite, arsenate, chromate, phosphate, selenite, selenate, borate, nitrate, *etc.*) and monoatomic anions (*e.g.* fluoride, chloride, bromide, and iodide) have been found in the environment and they can be harmful to both humans and wildlife.^1–3^ Some of these anions have become the most frequently detected in ground water at hazardous waste sites and have been placed on the top of the priority list of toxic pollutants by the U. S. EPA.^4^ Treatment of anion-containing wastewater prior to discharge is essential. Conventional techniques, such as reduction, reverse osmosis, electrodialysis, ion exchange, and adsorption, have been used for removing these anions from wastewater.^5,6^ However, the reduction followed by precipitation has some disadvantages, *i*.*e*., higher waste treatment equipment costs, significantly higher consumption of reagents, and significantly higher volume of sludge generated.^7^ Although reverse osmosis and electrodialysis are superior in recovering some of the oxyanions, such as Cr(vi), it is difficult to reduce the oxyanions in the effluent to an acceptable level.^6^ As far as ion exchange is concerned, it is an attractive approach in treating the wastewater containing anions, but ion exchange system is the complexity in regenerating the resin.^8^

Recently, Fe_3_O_4_-based magnetic nanoparticles (MNPs) have found to be simple, convenient, and powerful approaches for the separation and purification of environmental samples, and removal of toxic pollutants, including various ions, in water.^9–15^ However, most reports are focused on the treatment of one-ion component solution. Those for co-existing solutions, especially for adsorption mechanism investigating are quite limited.^5,9,11^ The industrial effluents often contain several kinds of oxyanions and monoatomic anions, the study of which is very complicated because of their synergistic, antagonistic or non-interactive effects on their adsorption. The traditional one-factor-at-a-time approach to run and analyze the experiments cannot successfully predict possible interactions between the oxyanions and monoatomic anions in industrial wastewater. Thus, it is necessary to investigate the simultaneous removal process in multi-component system containing oxyanions and monoatomic anions.

Multivariate analysis allows significant reduction in the number of experiments in addition to the description of independent variables impact on the process. This can contribute to the development and optimization of the multi-component system while it significantly decreases the cost of experiments. Response surface methodology (RSM) is of the most popular methods applied in researches on adsorption processes.^16,17^ The RSM is a useful statistical tool used to design experiments in which factors and their levels are determined. After handling the response of the experiments, the results are obtained by analyzing the response according to the RSM. A mathematical model is set *via* RSM by considering both linear and nonlinear relationships between independent variables, namely factors and response. If interactions affect the response, it can be mathematically modeled which allows for the optimization of the response. Based on such model, response surface graph and contours are provided, which help to visualize the shape of response surface.^18,19^ Thus, simultaneous modeling and optimization of variables are required to achieve an economic and popular wastewater treatment.

In this work, a tetraethylenepentamine (TEPA)-functionalized nano-Fe_3_O_4_ magnetic composite materials (nFe_3_O_4_@TEPA) was synthesized by a facile one-pot solvothermal method. It was characterized by elementary analysis (EA), powder X-ray diffraction (XRD), Fourier transform infrared spectrometer (FTIR), transmission electron microscopy (TEM), dynamic light scattering (DLS) and vibrating sample magnetometer (VSM).

**Scheme 1:**
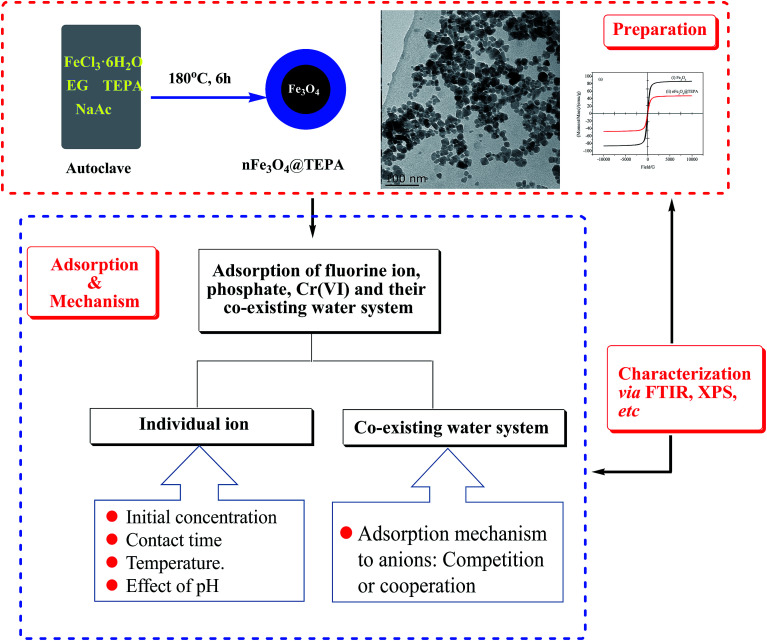
Overall procedure of the nFe_3_O_4_@TEPA facile preparation and its adsorption to anions.

The objective of this study is to investigate its adsorption properties of fluorine ion, phosphate, Cr(vi) and their co-existing water system. On the basis of the adsorption performance investigating of single-component for each ion, the adsorption of multi-component of the co-existing system was statistically studied. Presumed mechanisms were deeply investigated based on batch tests, thermodynamic and kinetic studies, XPS and FTIR characterization and RSM analyses. The overall procedure of the present work was shown in Scheme 1.

## 2 Experimental

### 2.1 Materials

Ferric chloride (FeCl_3_·6H_2_O), sodium acetate (NaAc), ethylene glycol (EG), potassium fluoride (KF), potassium dihydrogen phosphate (KH_2_PO_4_), potassium dichromate (K_2_Cr_2_O_7_) were analytical grade, and purchased from Sinopharm Chemical Reagent Co., Ltd. Tetraethylenepentamine (TEPA) was supplied by Aladdin Chemical Reagent Co., Ltd. (Shanghai, China). Distilled water was used to prepare all the solutions. 0.5 mol L^−1^ HNO_3_ and 0.5 mol L^−1^ NaOH solutions were used for pH adjustment.

### 2.2 Preparation of nFe_3_O_4_@TEPA

The overall preparation of nFe_3_O_4_@TEPA was produced using a polyol-media one-pot solvothermal method. 4.0 g of FeCl_3_·6H_2_O, and 12.0 g of NaAc were dissolved in 120 mL ethylene glycol. This solution was stirred vigorously at room temperature for 10 min to form a stable orange solution. 40 mL of TEPA was then added with constant stirring for 30 min until completely dissolved. The mixture solution was then transferred to a Teflon-lined autoclave and heated at 180 °C for 8 h. After the autoclave cooled to room temperature, the resulting nFe_3_O_4_@TEPA was isolated under magnetic field and washed with water and ethanol to remove redundant reagents and impurities. The as-prepared nFe_3_O_4_@TEPA was dried in a vacuum oven at 60 °C for 12 h and stored in a sealed bottle for further use.

### 2.3 Characterization

Transmission electron microscopy (TEM) images were obtained on a JEM-2100F Lorentz-Transmission Electron Microscopy (TEM) at an accelerating voltage of 200 kV. The magnetic properties of magnetic particles were measured using a vibrating sample magnetometer (VSM, Lake Shore 7410). Powder X-ray diffraction (XRD) patterns were collected on an X-ray diffractometer (Bruker D8 Advance) with CuKα radiation at *λ* = 0.154 nm operating at 40 kV and 40 mA. The elementary analysis results of the nitrogen contents in nFe_3_O_4_@TEPA were measured using an elementary analysis (EA, Thermo Fisher Flash-1112). Fourier Transform Infrared spectrometer (FTIR, Thermo Nicolet, USA) and X-ray photoelectron spectroscopy (XPS, AXIS ULTRADLD) were used to investigate the adsorption mechanism. Dynamic light scattering (DLS, Nano ZS-90) was used to determine the mean particle size.

The content of Fe_3_O_4_ in nFe_3_O_4_@TEPA was calculated from the amount of leached Fe, which was measured by using a spectrophotometer (722, Shanghai, China) according to the standard colorimetric method^11^ after digesting nFe_3_O_4_@TEPA in 12 mol L^−1^ HCl solution. The concentration of fluoride ion (F^−^), phosphate or Cr(vi) in the aqueous solution was analyzed following the standard methods for examination of water and wastewater.^20^ Briefly, the concentration of fluoride ion was carried out using combined fluoride-specific ion-selective electrode carried out using combined fluoride-specific ion-selective electrode on a SevenMulti™ instrument (Mettler Toledo). The concentration of phosphate was analyzed spectrophotometrically by the molybdenum blue method at 690 nm by adding (NH_4_)_6_Mo_7_O_24_ and SnCl_2_–HCl solutions followed by being kept in the dark for 10 min at room temperature (722, Shanghai, China). The concentration of Cr(vi) ions in the aqueous solution was analyzed with a spectrophotometer at a wavelength of 540 nm after acidification of samples with 1 N H_2_SO_4_ and reaction with 1,5-diphenyl carbazide to produce a purple color complex for colorimetric measurement (722, Shanghai, China).

### 2.4 Adsorption experiments

A stock solution of fluoride ion (F^−^), phosphate or Cr(vi) at concentration of 1000 mg L^−1^ was prepared by dissolving a known quantity of potassium fluoride (KF), potassium dihydrogen phosphate (KH_2_PO_4_) or potassium dichromate (K_2_Cr_2_O_7_) in ultrapure water. Batch adsorption experiments were carried out in 150 mL stoppered flasks, each of which contained 25.00 mL of fluoride ion (F^−^), phosphate or Cr(vi) individual solutions or co-existing solutions of varying concentration, from 10 to 1000 mg L^−1^. A 0.02 g amount of nFe_3_O_4_@TEPA was added into each flask and shaken at 150 rpm in a thermostatic shaker. 0.5 mol L^−1^ HNO_3_ and 0.5 mol L^−1^ NaOH solutions were used for pH adjustment, ranging from 2.0 to 10.0. Adsorption kinetic and thermodynamic studies at different temperatures (25–45 °C), with contacting time ranging from 1 to 180 min, were also carried out. Effect of co-existing ions was studied through Box-Behnken Design (BBD) and the response surface methodology (RSM). The post-adsorption solutions were separated magnetically under a NdFeB magnet.

According to the concentrations before and after adsorption, the equilibrium adsorption capacity (*q*, mg g^−1^) of the studied anions absorbed to the nFe_3_O_4_@TEPA is calculated using eqn (1):^21^1
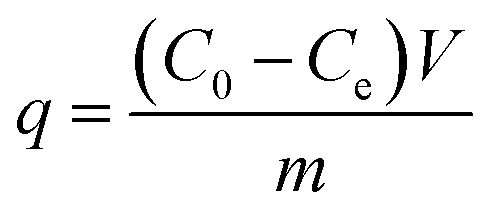
where *C*_0_ and *C*_e_ represent the initial solution concentration and the equilibrium concentration of fluoride ion (F^−^), phosphate or Cr(vi) (mg L^−1^), *V* is the volume of the solution (mL), *m* is the adsorbent dosage (mg), the same hereinafter.

For the kinetic studies, the pseudo-first-order model (eqn (2)),^21^ pseudo-second-order model (eqn (3)),^21,22^ and intraparticle diffusion model (eqn (4)),^21^ were used to fit the experimental data.2
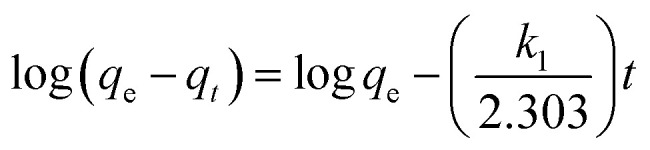
3
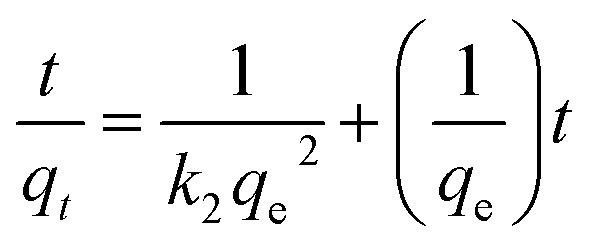

4 *q*_*t*_ = *k*_id_*t*^1/2^ + *C*

where, *q*_e_ and *q*_*t*_ are the adsorption capacities at equilibrium and at time *t* (mg g^−1^), respectively. *k*_1_ (min^−1^), *k*_2_ (g (mg^−1^ min^−1^)) are the adsorption rate constants, *k*_id_ is the intraparticle diffusion rate constant (mg (g^−1^ min^−1/2^)), *C* is the intercept (mg g^−1^).

For the adsorption isotherm studies, two adsorption isotherms, Langmuir model (eqn (5))^21,23^ and Freundlich model (eqn (6)) were applied to analyze the adsorption data.^21,24^5
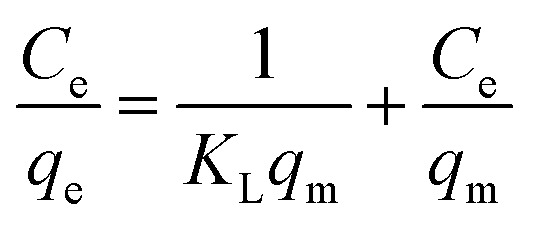

6 log *q*_e_ = log *K*_F_ + (1/*n*)log *C*_e_

where *q*_m_ and *K*_L_ are the Langmuir constants related to the maximum adsorption capacity and apparent heat change, respectively, while *K*_F_ is a Freundlich constant related to adsorption capacity and 1/*n* is a Freundlich constant related to the adsorption intensity.

## 3 Results and discussion

### 3.1 Characterization of nFe_3_O_4_@TEPA

**Fig. 1:**
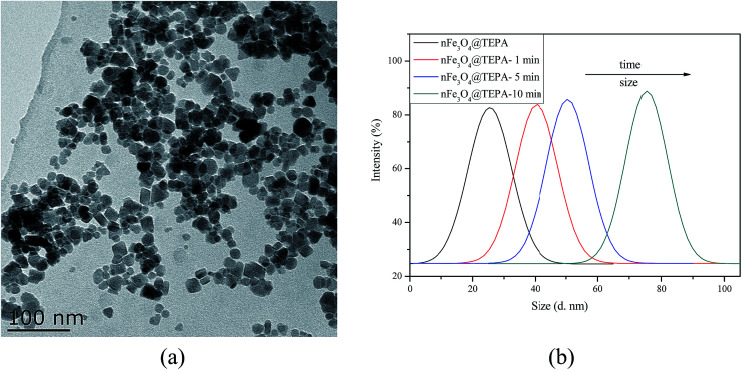
TEM image (a) and DLS (b) of nFe_3_O_4_@TEPA.

The TEM images of nFe_3_O_4_@TEPA were shown in Fig. 1(a). All the size data reflect the averages of about 100 particles and are calculated according to eqn (7):^25^7
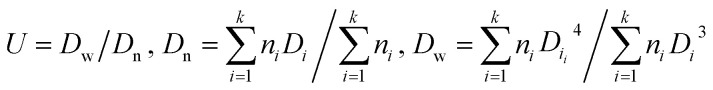
where *U* is the polydispersity index, *D*_n_ is the number-average diameter, *D*_w_ is the weight-average diameter, and *D*_*i*_ is the diameter of the determined microspheres. It revealed that the nFe_3_O_4_@TEPA particles were multidispersed with some aggregation at an average diameter of around 20 nm (Fig. 1(a)), with *D*_n_ at 21.5, *D*_w_ at 22.8, and *U* at 1.06. In order to check the aggregation behavior, we carried out the DLS experiments of nFe_3_O_4_@TEPA from 0–10 min. As shown in Fig. 1(b), the particles aggregated gradually and the intensity average diameter measured by DLS increased from 25 nm to 40 nm after 1 minute, and 75 nm after 10 minutes. This might be due to the dipolar magnetic interaction between the magnetic cores and hydrogen bonds between the amino groups on the surface of the magnetic cores.

**Fig. 2:**
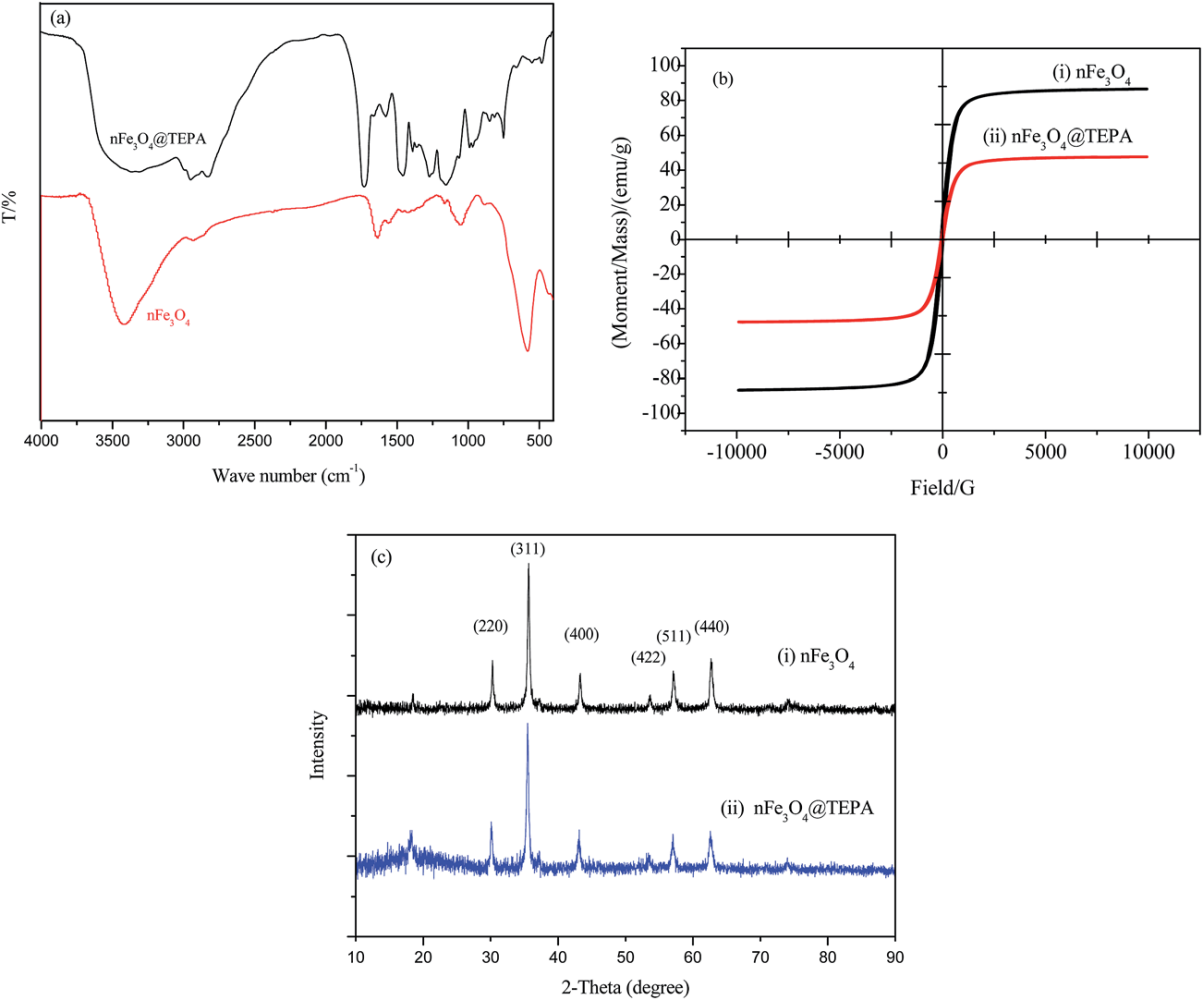
FTIR (a), VSM (b) and XRD (c) of nFe_3_O_4_ and nFe_3_O_4_@TEPA

The FTIR spectra of nFe_3_O_4_ and nFe_3_O_4_@TEPA were showed in Fig. 2(a). Characteristic band of nFe_3_O_4_ occurs at ∼589 cm^−1^. Other typical bands can be assigned as follows, *υ*(–OH): ∼3446 cm^−1^, *υ*(–CH_2_): ∼2924 cm^−1^, ∼2853 cm^−1^, *δ*(–CH_2_): ∼1429 cm^−1^ for PEG. Compared with nFe_3_O_4,_ After functionalization by TEPA, typical bands at ∼1573 cm^−1^ can be assigned to be the stretching and bending vibrations of the –NH and –NH_2_ groups appeared, with a great shift of the bands for *δ*(–CH_2_). This revealed that the amino groups of TEPA had been successfully grafted to the surface of the nFe_3_O_4_. The superparamagnetic properties of the nFe_3_O_4_@TEPA were verified by the magnetization curve measured by VSM, shown in Fig. 2(b). The saturation moment obtained from the hysteresis loop was found to be 48.2 emu g^−1^. The nFe_3_O_4_@TEPA was expected to respond well to magnetic fields without any permanent magnetization, therefore making the solid and liquid phases separate easily. Due to the anti-magnetic property of the TEPA, it was no surprise the saturation moment of nFe_3_O_4_@TEPA lower than that of the naked nano-Fe_3_O_4_ (78.6 emu g^−1^, as shown in Fig. 2(b)). Interestingly, the saturation moment of the present nFe_3_O_4_@TEPA was much higher than those of our previously reported nano magnetic polymers (NMPs), which was ranged from 12.3 to 5.56 emu g^−1^,^26^ which might be due to two facts: (1) anti-magnetic polymer anchored onto the Fe_3_O_4_ core of the NMPs, which leading a decrease of content percentage of Fe_3_O_4_ in the NMPs; (2) by using solvothermal method, amino-groups of TEPA self-assembled graft to the surface of the magnetic cores *via* hydrogen bonds between the amino groups and active hydroxyl groups of Fe_3_O_4_. The obtained nFe_3_O_4_@TEPA is with good dispersity to avoid the dispersion agglomeration defects in the traditional preparation process. With a large number of amino groups on the surface of the nFe_3_O_4_, it is beneficial to form magnetic ordered structure, which leading an increase of the saturation moment. This phenomenon was also found by Yoon, *et al.*^27^ High saturation magnetization (56.1 emu g^−1^) of the Fe_3_O_4_ based magnetic polymer composite material-Fe_3_O_4_@DAPF was found when solvothermal method was used for preparation. To further demonstrate the crystal structure of nFe_3_O_4_@TEPA, the XRD patterns of the as-prepared Fe_3_O_4_ (without adding TEPA) and nFe_3_O_4_@TEPA were collected (Fig. 2(c)). It indicated that nFe_3_O_4_@TEPA had retained the spinel structure of Fe_3_O_4_, in which the identical peaks for Fe_3_O_4_ located at 30.1°, 35.5°, 43.1°, 53.4°, 57.0° and 62.6°, corresponding to their indices (220), (311), (400), (422), (511) and (400) appeared.^28^

Elemental analysis results showed that nitrogen percentage of nFe_3_O_4_@TEPA was 18.9%, while the total content of Fe_3_O_4_ in the nFe_3_O_4_@TEPA was 58.2%, which was higher than those of our previously reported NMPs, and consisted with the VSM results.

### 3.2 Adsorption mechanism of the nFe_3_O_4_@TEPA to anions

#### 3.2.1 Effect of pH and adsorption mechanism for the individual ion

**Fig. 3:**
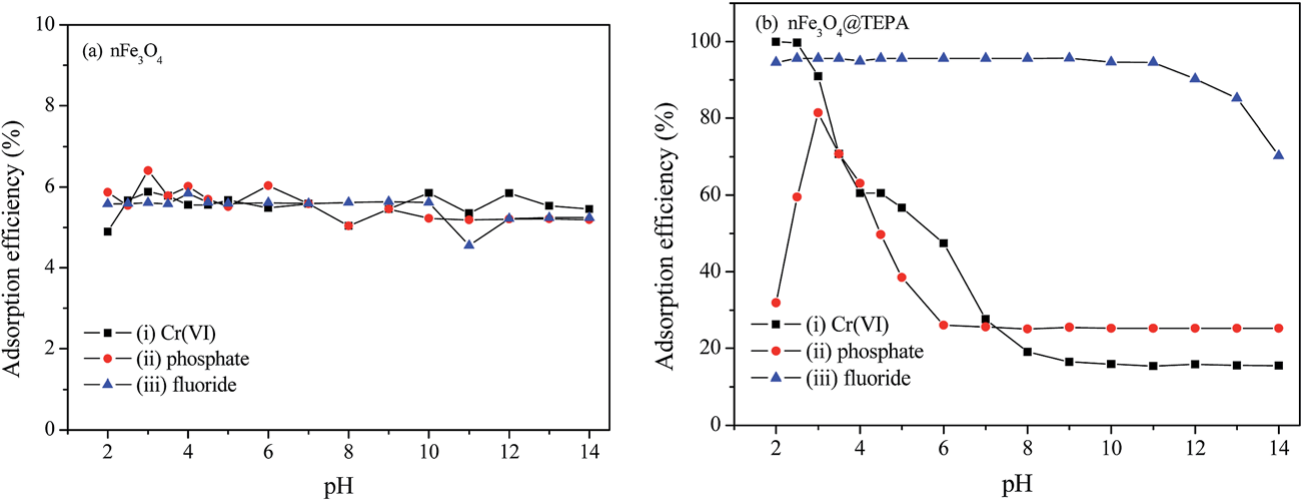
The pH effect on the adsorption of fluoride ion (F^−^), phosphate or Cr(vi) individual solutions at concentration of 50 mg L^−1^ of nFe_3_O_4_ (a) and nFe_3_O_4_@TEPA (b).

The pH effect of fluoride ion (F^−^), phosphate or Cr(vi) individual solutions at concentration of 50 mg L^−1^, respectively, was investigated with the pH values ranging from 2.0 to 10.0, and the results were shown in Fig. 3. For nFe_3_O_4_, the adsorption efficiencies of all the three ions were quite low (at around 5%) and almost not dependent on solution pH, shown in Fig. 3(a). However, the adsorption efficiencies of nFe_3_O_4_@TEPA were much higher than those of nFe_3_O_4_, at 15.5–99.9% depending on different ions and pH values, shown in Fig. 3(b). The high efficiencies might be contributed to the amino-groups of TEPA anchored on the surface of the nFe_3_O_4._ The adsorption efficiency of phosphate or Cr(vi) was highly dependent on solution pH. Interestingly, unlike phosphate and Cr(vi), the adsorption capacity of fluoride ion (F^−^) was almost not dependent on solution pH. These results imply there might be different adsorption mechanism of nFe_3_O_4_@TEPA to these three kinds of anions.

**Fig. 4:**
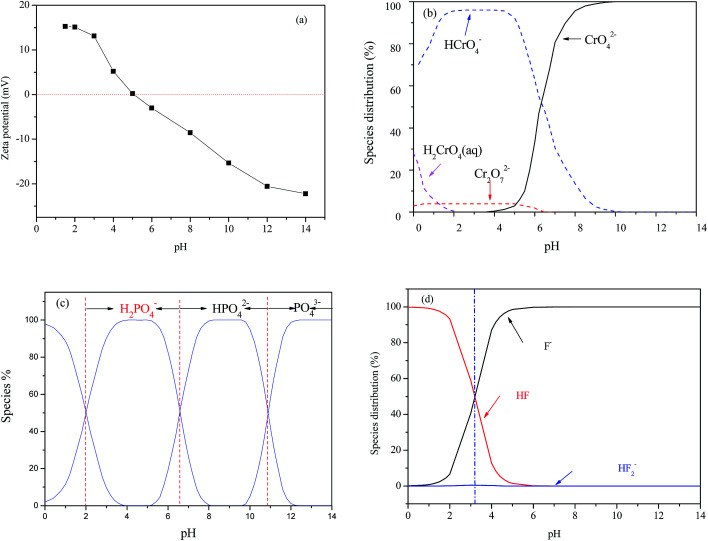
pH_pzc_ of the nFe_3_O_4_@TEPA (a); distribution diagrams of Cr(vi) (b), phosphate (c) and fluorine (d) species.

The pH dependency might be related both to the intrinsic structure property of the nFe_3_O_4_@TEPA and the species of anions. Fig. 4(a) showed that the pH_pzc_ of the nFe_3_O_4_@TEPA was identified to be 5.02, implying the outer surface of the nFe_3_O_4_@TEPA is positively charged when pH is below 5.02, and negatively charged when pH is above 5.02. Based on the experimental data of the total concentration, we run Visual MINTEQ 3, which is widely used in recent years to simulate equilibria and speciation of inorganic solutes in aqueous solution.^29–32^ The speciation of Cr(vi), phosphate and fluorine under various pH was obtained, as shown in Fig. 4(b)–(d).

In the case of Cr(vi), as shown in Fig. 3, the percentage of uptake Cr(vi) for nFe_3_O_4_@TEPA decreased from 99.9% to 16.8% gradually with an increase of pH value from 2.0 to 10.0. This phenomenon is contributed to the pH-dependent adsorption mechanism. As shown in Fig. 4(b), five main pH-dependent species of Cr(vi) exist, *i.e.,* H_2_CrO_4_, HCrO_4_^−^, CrO_4_^2−^, HCr_2_O_7_^−^, Cr_2_O_7_^2−^, as described by eqn 8–11. Cr(vi) exists mainly in the soluble form of H_2_CrO_4_ at pH less than 2.0, HCrO_4_^−^ with pH from 2.0 to 4.0, and CrO_4_^2−^ at pH value above 6.5.8
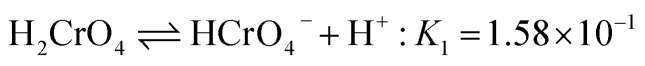
9
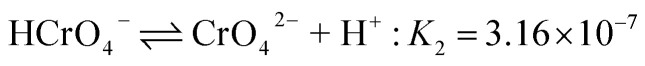
10
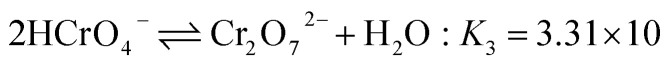
11
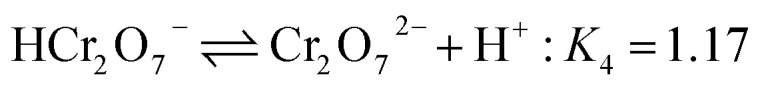


Under acidic conditions (pH at 2.0–3.5), amino groups were easier to be protonated (–NH_3_^+^), as described by eqn (12). Electrostatic attraction happened as in eqn (13),^33^ leading a decrease of the residue concentration of Cr(vi). With increasing of the pH value, the concentration of H^+^ was decreased, and at the same time the concentration of OH^−^, which competed with HCrO_4_^−^, was increased. So the ability of –NH_2_ to be protonated was weakened, resulting in the decline of removal efficiency.12
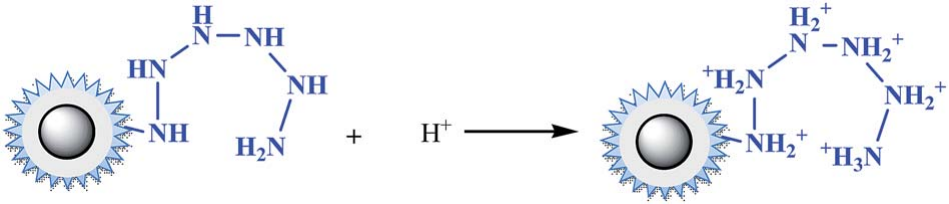
13
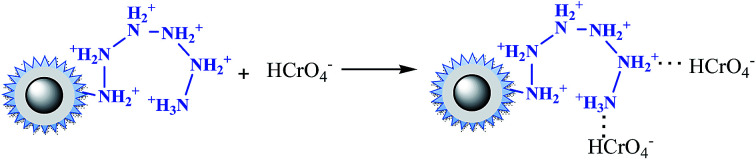


With the increasing of pH value, an interesting phenomenon was observed that there was a flat, as we found before in amino-functionalized nano magnetic polymers (NMPs).^26^ This implied that besides the electrostatic attraction and ion exchange interactions, coordination interactions might occur in the adsorption process. as in eqn (14).14
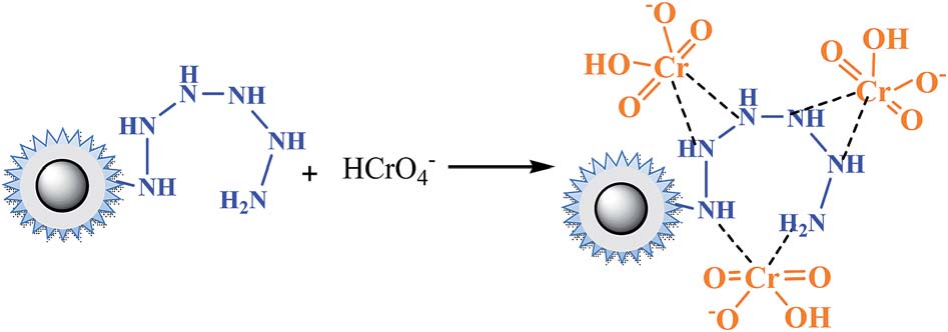


In the case of phosphate, as shown in Fig. 3(c), the percentage of uptake phosphate for nFe_3_O_4_@TEPA increased from 31.9% to 81.4% sharply to a maximum at pH 3.0, then decreased sharply to 26.0% at pH 6.0. As shown in Fig. 4(b), phosphate existed in the forms of H_3_PO_4_, H_2_PO_4_^−^, HPO_4_^2−^ and PO_4_^3−^, depending on the solution pH (p*K*_1_ = 2.15, p*K*_2_ = 7.20, and p*K*_3_ = 12.33).^34,35^ With an increase of pH, the nFe_3_O_4_@TEPA surface carried positive charge, and thus would more significantly attract the negatively charged monovalent H_2_PO_4_^−^ ions in solution, which indicated that the physicochemical adsorption due to electrostatic attraction was the predominant process of phosphate removal, as described by eqn (15). When the pH of the solution increased, the surface became negatively charged, consequently, unfavorable to the phosphate for electrostatic repulsion.15
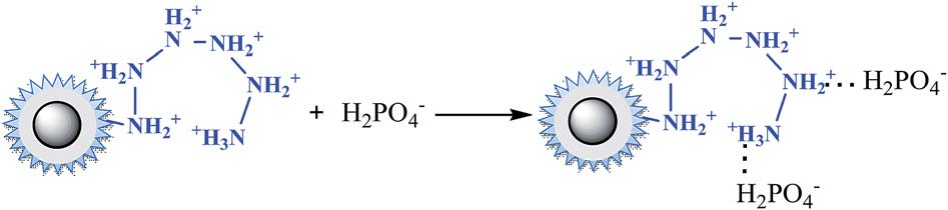


In the case of fluoride, as shown in Fig. 3(d), unlike Cr(vi) and phosphate, the adsorption capacity was almost not dependent on solution pH. To our knowledge, this is one of the longest pH ranges of the materials for F^−^ adsorption in literature. The percentage of uptake fluoride for nFe_3_O_4_@TEPA kept constant at around 95.6% under pH value from 2.0 to 11.0, and gradually decreased to 70.2% at pH 14. Such high fluoride removal efficiencies are much better than those in prior reports. For instance, Kong *et al.* reported the fluoride removal efficiency of MHS-MgO/MgCO_3_ adsorbent was 86.2%, 83.2% and 76.5% at pH = 11 for initial fluoride concentrations of 10, 20 and 30 mg L^−1^, respectively.^36^ Mohapatra *et al.* studied the fluoride removal efficiency of Mg-doped nano Fe_2_O_3_ adsorbent was almost 30% at pH = 11 initial fluoride concentration 10 mg L^−1^.^37^ Swain *et al.* demonstrated that the fluoride removal efficiency of Fe(iii)–Zr(iv) binary mixed oxide was about 38% at pH = 11.^38^ As shown in Fig. 4(c), fluoride mainly existed in the forms of HF, F^−^, depending on the solution pH (p*K* = 3.18). Although, some literatures^38,39^ showed that in the acidic pH range (pH < 5), weak hydrofluoric acid (HF) is present in the experiments may affect defluoridation, no obvious decrease of the uptake fluoride was observed in this work. Thus, we assume that besides electrostatic attraction, hydrogen bonds might form in the process of fluoride removal, as described by eqn (16)–(18). In the strongly alkaline range (pH > 11), there is a drop at around 25% in adsorption percentage, which may be due to the competition of hydroxyl ions with the fluoride and the electrostatic repulsion from the surface of nFe_3_O_4_@TEPA.16
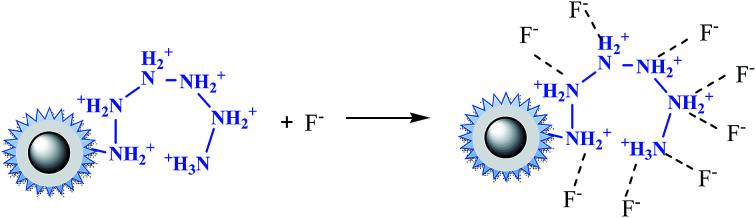
17
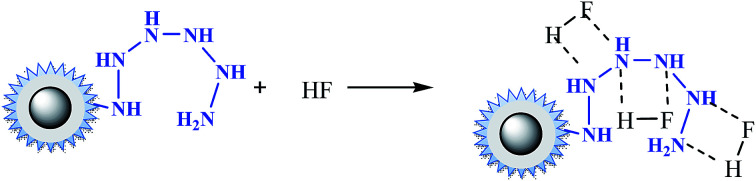
18
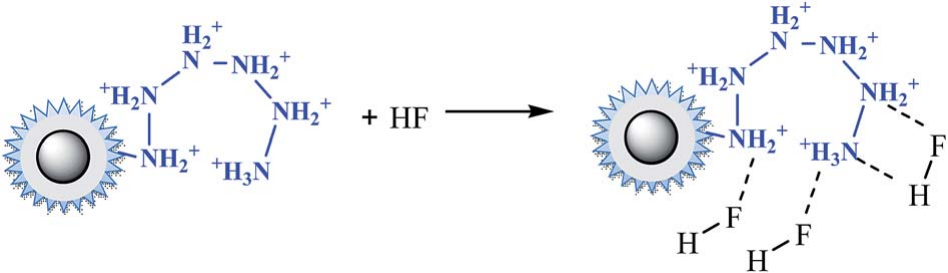


#### 3.2.2 Kinetic studies

Adsorption kinetics of mono-component fluoride ion (F^−^), phosphate or Cr(vi) onto nFe_3_O_4_@TEPA showed that all the three kinds of anions, the adsorption capacity increased rapidly and reached equilibrium in 10 min and intra-particle process might not be involved in the rate-limiting steps. The adsorption kinetic experimental data fit the pseudo-second-order model well for all the studied anions. The activation energies of the adsorption process, *E*_a_, are found to 19.56 kJ mol^−1^, 23.71 kJ mol^−1^, 25.35 kJ mol^−1^ for fluoride ion (F^−^), phosphate or Cr(vi), respectively. All are less than 42 kJ mol^−1^, indicating that diffusion process was the rate-controlled step.^40^ Detailed discussions were presented in ESI, S1 Kinetic studies and Fig. S1, Table S1 and S2.†

#### 3.2.3 Adsorption capacity of nFe_3_O_4_@TEPA to anions

The adsorption capacities of nFe_3_O_4_@TEPA to fluoride ion (F^−^), phosphate or Cr(vi) mono-component were investigated. Detailed discussions were presented in ESI, S2 Adsorption capacity and Fig. S2, Table S3 and S4.† The results showed that the Langmuir models fit the data well, suggesting a monolayer adsorption. The maximum adsorption capacities (*q*_m_) for fluoride ion (F^−^), phosphate or Cr(vi) are 163.9, 149.3, and 400 mg L^−1^, respectively. Interestingly, although the adsorption to all the studied anions the nFe_3_O_4_@TEPA was spontaneous in nature (Δ*G*^θ^ < 0), the enthalpy changes (Δ*H*^θ^) for the fluoride ion (F^−^), phosphate or Cr(vi) were found to be at 38.54, 13.89, 88.03 kJ mol^−1^ (Table S4†), respectively, which indicated that the adsorption was endothermic. For physical adsorption, the process of adsorption is usually exothermic, that is, the increase of temperature is not favorable to the adsorption. However, chemisorption is some of endothermic, and some of exothermic. In general, it is thought that the increase in temperature is beneficial to chemisorption.^41^ Similar results were found in our previous work^13,17^ and in literature.^41^

#### 3.2.4 Effect of co-existing ions and presumed mechanism

The effect of co-existing ions experimental studies were investigated with a standard response surface methodology (RSM) design called Box-Behnken Design (BBD). RSM is a useful mathematical and statistical technique for the development of empirical relation between the experimental outputs (responses) and process parameters (factors). A well designed RSM approach leads to optimize the process parameters for improving the responses. The experimental parameters (*X*_1_ (initial concentration of Cr(vi), C(Cr(vi))), *X*_2_ (initial concentration of fluoride ion (F^−^), C(F)), and *X*_3_ (initial concentration of phosphate, C(P)), for design of experiment strategy are considered at three levels and coded as −1, 0, and +1 for low, middle and high level respectively. The coded and actual values of the independent variables and predicted response of the model were shown in Table S5 and S6.† In the BBD modeling of three factors and three levels, the center point was repeated for five times in order to improve the accuracy in estimation of errors. The response of the model was analyzed by analysis of variance (ANOVA) and a second-order polynomial model (as shown in eqn (19)) was fitted to correlate between the independent variables (*X*_1_, *X*_2_ and *X*_3_) and the response for anions removal in order to predict the of co-existing ions effect.19
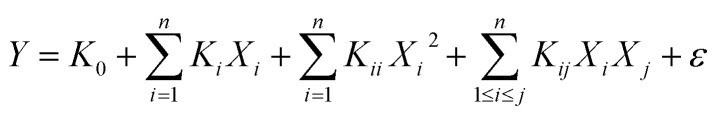
where *Y* represents the predicted response variables *i.e.* the amount of anions adsorbed by nFe_3_O_4_@TEPA, *K*_0_ is the constant coefficient, *K*_*i*_ is the linear coefficient of the input factor *X*_*i*_, *K*_*ii*_ is the ith quadratic coefficient of the input factors *X*_*i*_, *K*_*ij*_ is the different interaction coefficients between input factors *X*_*i*_ and *X*_*j*_, and *ε* is the error of the model. The software Design Expert (Version 8.0.6.1) was used for model statistic, like experimental design, determination of the coefficients, data analysis and the graph plotting.

The adsorption capacities of nFe_3_O_4_@TEPA to fluoride ion (F^−^), phosphate or Cr(vi) multi-component solution was carried out by means of BBD of RSM. Quadratic model were used to know the adsorption capacity of the fluoride ion (F^−^), phosphate or Cr(vi), respectively, shown in ESI eqn (S6)–(S8).† The positive sign and the negative sign of the term indicates the synergetic and antagonistic effect respectively. The ANOVA data shown in Table S7–S9 of ESI† for Response 1 (q(Cr)), Response 2 (q(F)), and Response 2 (q(P)), respectively. The coefficient of determination (*R*^2^), which measure the degree of fit in the model was found to be 0.9956, 0.9923, 0.9920 and an Adj-*R*^2^ of 0.9900, 0.9824, 0.9817, respectively. In addition, the model is very significant as evident from its *F*-value and very low probability *p*-value. If the *p*-value is less than 0.05, it indicates that the model is statistically significant whereas a value higher than 0.05 suggests that the model is not significant.^42,43^ Here, the *F*-values were found to be 177.03, 100.01, 96.19, respectively, and *p*-value were all < 0.0001 for the model.

Values of “Prob > *F*”less than 0.0500 indicate model terms are significant. As shown in the ANOVA data in Table S7–S9,† for the adsorption of Cr(vi), the linear terms, C(Cr(vi)) and C(P) are significant; while for adsorption of fluoride ion (F^−^), besides the linear terms, C(F), and C(P), quadratic terms of C(F),^2^ and one cross-product coefficients C(Cr(vi))C(F) and C(F)C(P) are significant; for adsorption of phosphate, except the one cross-product coefficients C(F)C(P) is not significant, all the other model terms are significant.

**Fig. 5:**
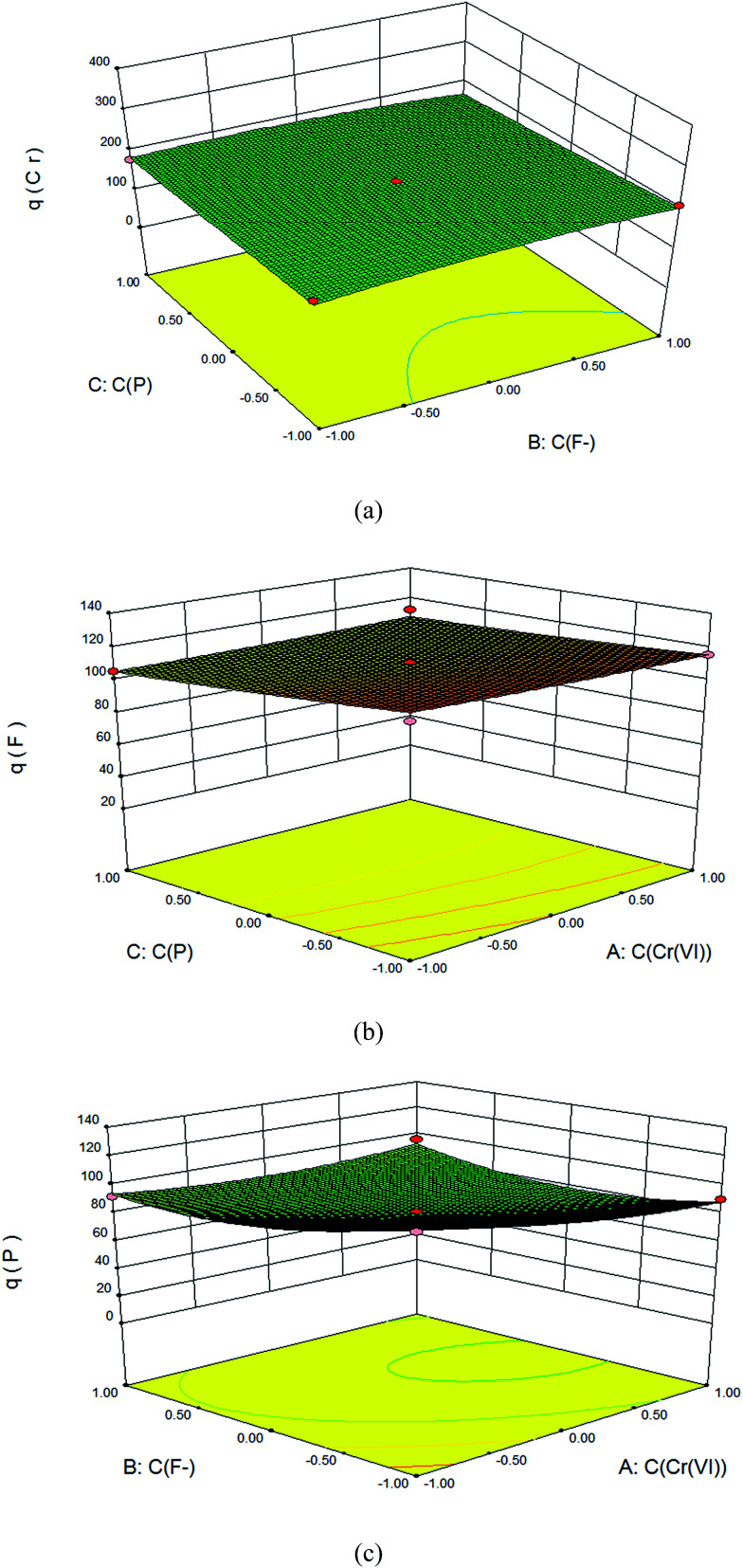
The effect of the co-existing fluoride ion and phosphate to the adsorption of Cr(vi) (a), the co-existing Cr(vi) and phosphate to the adsorption of fluoride ion (b), the co-existing Cr(vi) and fluoride ion to the adsorption of phosphate (c).

In response surface plots, the adsorption of anions can be better explained by the interaction of all the three factors. The three dimensional plots and contour plots were used to know the effect of two parameters in their experimental range for the removal of anions while the third parameter remains at zero level. From the shape of contour plot, one could be able to explain the nature and extents of interaction between the experimental factors, *i.e.*, the effects of the co-existing ions in the present work. Circular and elliptical shape of contour plots shows the significant interaction between the experimental factors in the model. Therefore maximum adsorption capacity can be explain on the basics of these experimental factors, *i.e.*, the effects of the co-existing ions, here. The effect of the co-existing fluoride ion (F^−^) and phosphate to the adsorption of Cr(vi) was shown in Fig. 5(a), while the effect of the co-existing Cr(vi) and phosphate to the adsorption of fluoride ion was shown in Fig. 5(b) and the effect of the co-existing Cr(vi) and fluoride ion to the adsorption of phosphate was shown in Fig. 5(c), respectively. Those of the bi-component systems were shown in the Fig. S3 in the ESI.† As shown in Fig. 5, for the tri-component co-existing system, either fluorine ion or phosphate, had little effect on the adsorption competition to Cr(vi). Cr(vi) took priority for adsorption and could replace the absorbed fluorine ion or phosphate by competitive reaction. As shown in Fig. S3,† for the fluorine ion and phosphate bi-component system, the adsorption of them was competitive *via* ion exchange.

**Fig. 6:**
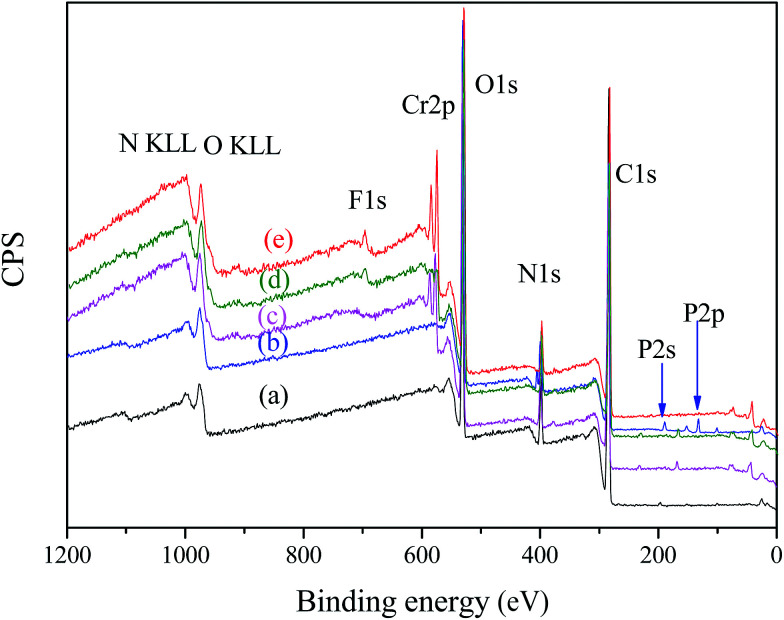
XPS spectra of nFe_3_O_4_@TEPA before adsorption (a), after adsorption of phosphate (b), after adsorption of Cr(vi) (c), after adsorption of fluoride ion (d) and after adsorption of the co-existing the three anions (e).

The adsorption mechanism could be confirmed by XPS and FTIR analyses of nFe_3_O_4_@TEPA before and after adsorption of the studied anions. The survey scan of XPS spectra of nFe_3_O_4_@TEPA before (a) and after adsorption of phosphate (b) Cr(vi) (c), fluoride ion (d) and the co-existing the three anions (e) were shown in Fig. 6. From the survey scan of XPS spectra (Fig. 6), new peaks owing to P2p, Cr2p and F1s appeared after adsorption of phosphate (b), after adsorption of Cr(vi) (c), after adsorption of fluoride ion (d) and after adsorption of the co-existing the three anions (e), suggesting that the phosphate, Cr(vi) and fluoride ion were successfully adsorbed on the surface of nFe_3_O_4_@TEPA. High-resolution XPS spectra of nFe_3_O_4_@TEPA after adsorption of the co-existing the three anions were shown in Fig. S4.† As shown in Fig. S4(a),† the characteristic peaks for Cr(vi) (Cr2p 1/2, 587.5 eV; Cr2p 3/2, 579.4 eV) appeared, no obvious peaks assigned to Cr(iii), normally appeared at Cr2p 1/2, 586.3 eV; Cr2p 3/2, 577.1 eV, were observed,^44,45^ which implied that the main species existed in the surface of the nFe_3_O_4_@TEPA was Cr(vi), reduction to Cr(iii) hardly occurred, differing from our previous founding.^12^ The characteristic peaks for phosphate (P2p, 113.0 eV) and fluoride (F1s, 685.2 eV) can be found in Fig. S4(b) and (c),† respectively, which clearly confirmed the successful adsorption of phosphate^46^ and fluoride.^47^ As shown in Fig. S4(d),† after adsorption, the peaks of N1s appeared at 398.8 eV with a broader band range, which could be attributed to protonated amine groups (–NH_3_^+^) and the further formation of –NH_3_^+^⋯anions.^48^ Similar phenomena were observed in the XPS spectra of O1s (Fig. 4(e)), peaks of O1s appeared at ∼531.1 eV and ∼529.5 eV, assigned to C–O–C and C–OH groups, broadening with a slight shift of binding energies. In the XPS spectra of C1s (Fig. 4(f)), the carbon atoms can be found in two chemically different positions, leading to two differing C1s binding energies: C–O–C (∼282.6 eV) and C–O–C (∼284.0 eV) or C–OH (∼286.5 eV). Changes in atomic concentration of the key elements after the adsorption were summarized in Table S10.† The main elements of the nFe_3_O_4_@TEPA were Fe, O, N and C. Compared with the initial pre-adsorbed material, the chromium, phosphate, and fluoride atomic percent of the sample was 1.88%, 1.01% and 0.91% after adsorption experiment. It confirmed that the studied anions were undoubtedly adsorbed onto the surface of nFe_3_O_4_@TEPA.

**Fig. 7:**
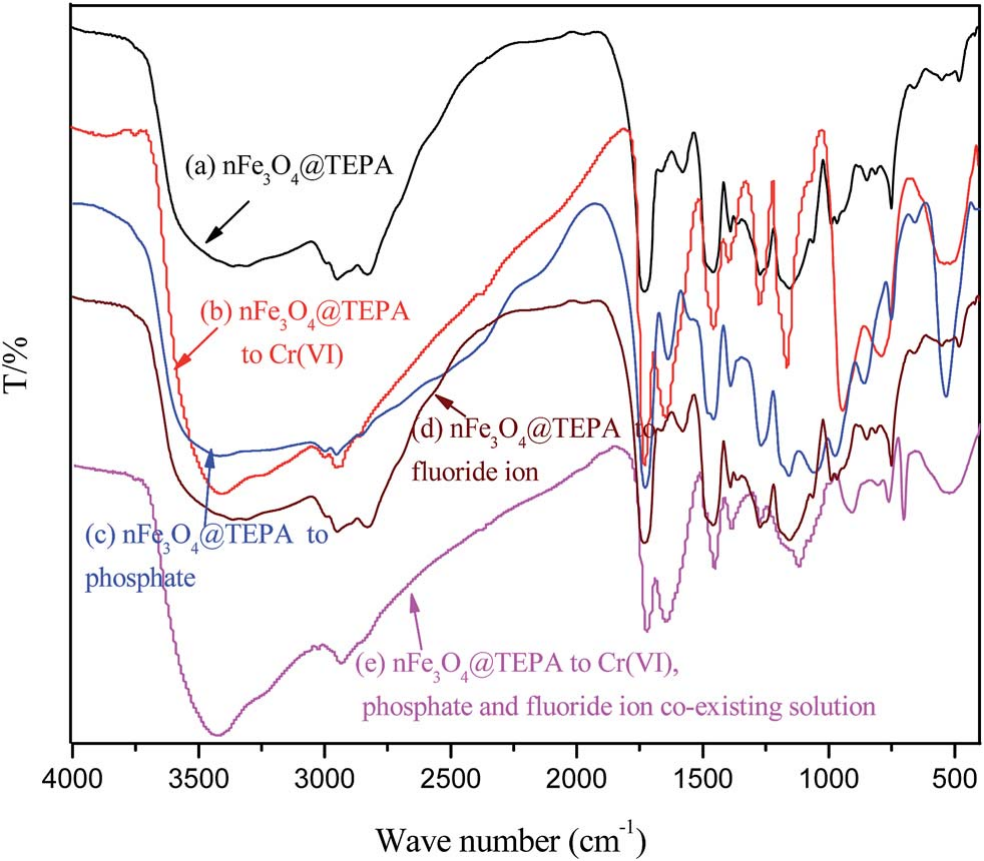
FTIR spectra of nFe_3_O_4_@TEPA before adsorption (a), after adsorption of phosphate (b), after adsorption of Cr(vi) (c), after adsorption of fluoride ion (d) and after adsorption of the co-existing the three anions (e).

The FTIR spectra of nFe_3_O_4_@TEPA before (a) and after adsorption of phosphate (b), Cr(vi) (c), fluoride ion (d) and the co-existing the three anions (e) were showed in Fig. 7. In Fig. 7(a), the broad peak appeared at ∼3360 cm^−1^ and ∼1573 cm^−1^ can be assigned to be the stretching and bending vibrations of the –NH and –NH_2_ groups. While after adsorption, in Fig. 7(b)–(e) the characteristic bands at ∼1573 cm^−1^ disappeared along with the appearance of the bands at ∼1630 cm^−1^, which may be attributed to the interaction between amino groups and the phosphate, Cr(vi) and fluoride groups, subsequently weakened the N–H bonding and resulted in a large shift (∼80 cm^−1^). The characteristic peaks of Cr(vi) at ∼940 cm^−1^ and ∼760 cm^−1^can be observed in the absorption of HCrO_4_^−^ and the typical peak at ∼540 cm^−1^ for the “Cr–N” also appeared as shown in Fig. 7(b) and (e). The characteristic peaks of the phosphate groups at 543 cm^−1^ were also observed in Fig. 7(c) and (e), corresponding to the –P–O and –O–P–O groups, respectively.

#### 3.2.5 Reusability investigation

**Fig. 8:**
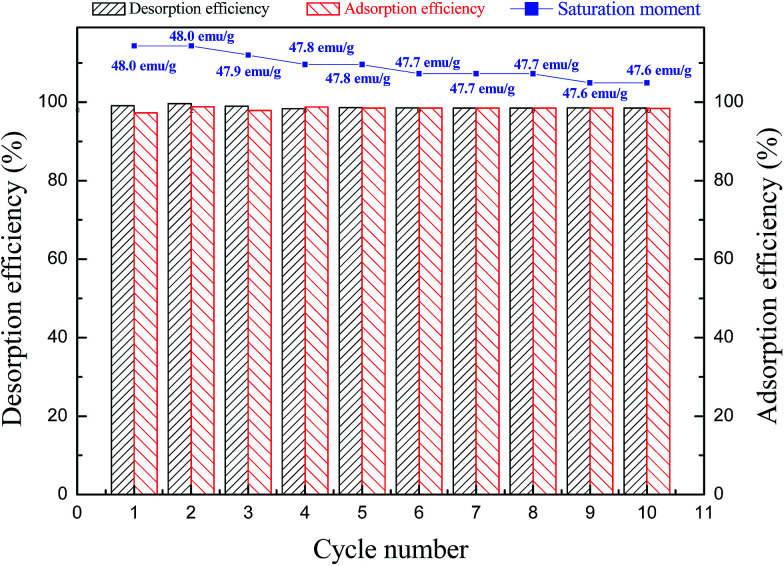
Reusability of nFe_3_O_4_@TEPA after adsorption of a mixture solution of fluoride ion (F^−^), phosphate and Cr(vi) at each concentration at 20 mg L^−1^ (insert: saturation moments of the reused nFe_3_O_4_@TEPA from 1 cycle to 10 cycles).

The reusable of the nFe_3_O_4_@TEPA was evaluated by comparing the average adsorption efficiency of a mixture solution of fluoride ion (F^−^), phosphate and Cr(vi) at each concentration at 20 mg L^−1^. The post-absorbed nFe_3_O_4_@TEPA was extracted with 1% NaOH methanol solution for 30 min, and for another adsorption to get the next adsorption efficiency. The results were shown in Fig. 8, indicating that nFe_3_O_4_@TEPA could be used for at least 10 cycles with a loss of less than 2.8% upon recovery on average. No obvious decrease in the adsorption capacity efficiency was found. The VSM experiments of the recycled nFe_3_O_4_@TEPA were tested. The saturation moments obtained from the hysteresis loops were found to be 48.0–47.6 emu g^−1^ from 1 cycle to 10 cycles, (as shown in Fig. 8 (insert)). Compared with the saturation moment of the fresh-prepared nFe_3_O_4_@TEPA (48.2 emu g^−1^), which implied that no obvious decrease reduction of the magnetic strength was found.

## 4 Conclusion

A tetraethylenepentamine (TEPA)-functionalized nano-Fe_3_O_4_ magnetic composite materials (nFe_3_O_4_@TEPA) was synthesized by a facile one-pot solvothermal method. The as-prepared nFe_3_O_4_@TEPA exhibited a homogeneous morphology, strong affinity ability, and high magnetic responsiveness for the adsorption of ions. The adsorption of the multi-ion co-existing system was studied *via* batch tests, XPS and FTIR analyses, and analyzed *via* response surface methodology (RSM). The adsorption mechanism of multi-ion component system was intensively studied.

## Conflicts of interest

There are no conflicts to declare.

## Supplementary Material

RA-008-C8RA00237A-s001
