# Supplementary material for: Facile preparation of a tetraethylenepentamine-functionalized nano magnetic composite material and its adsorption mechanism to anions: competition or cooperation
Source: RSC Adv. 2018 Mar 16;8(19):10686–97. doi: 10.1039/c8ra00237a (PMC9078924; doi:10.1039/c8ra00237a)
Supplement: RA-008-C8RA00237A-s001 [file RA-008-C8RA00237A-s001.pdf]

Electronic Supplementary Material (ESI) for RSC Advances.
This journal is © The Royal Society of Chemistry 2018

Electronic Supplementary Material (ESI)

Facile preparation of tetraethylenepentamine-functionalized nanomagnetic composite material and its adsorption mechanism to anions: Competition or cooperation

Meiqin Hu, Haoyu Shen*, Si Ye, Yan Wang, Jiali Zhang, Shanshan Lv
Ningbo Institute of Technology, Zhejiang University; Ningbo, Zhejiang, 315100, China

S1. Kinetic studies

Fig. S1 presented the adsorption kinetics of mono-component fluoride ion (F^-), phosphate or Cr(VI) onto nFe_3O_4@TEPA. As shown in Fig. S1(a), for all the three kinds of anions, the adsorption capacity increased rapidly and reached equilibrium in 10 min. This revealed that the presence of surface grafting of amino groupson adsorbents could effectively shorten the equilibrium time. Besides, the kinetic curve of nFe_3O_4@TEPAcould be divided into two portions, which could be described by intraparticle diffusion model (shown in Fig. S1(b)) and indicated that the intra-particle process[1] might not be involved in the rate-limiting steps. Therefore, in the present case, anions reached the nFe_3O_4@TEPAeasily and took less time to reach adsorption equilibrium, implying that the surface grafting of amino groups and uniform structures of nFe_3O_4@TEPA allowed efficient mass transport, thus overcoming some drawbacks of traditionally materials.

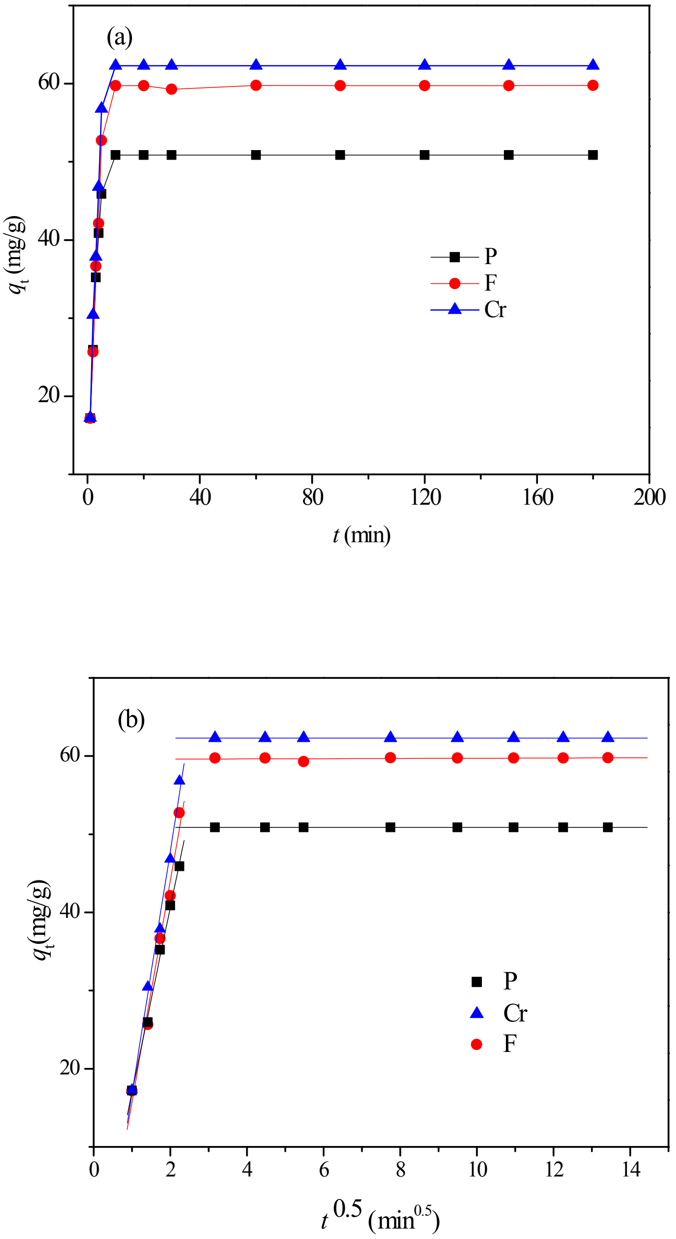

Fig. S1 Effect of adsorption time on the adsorption of mono-componentfluoride ion (F^-), phosphate or Cr(VI) onto nFe_3O_4@TEPA

Pseudo-first-order and pseudo-second-order models were used to describe the adsorption kinetic data. The correlation coefficient values indicated a better fit of the pseudo-second-order model with the experimental data compared to the pseudo-first-order for all the studied anions. The equations and constants, including k_2, $q_{e,cal}$, and initial adsorption rates ($k_2q_e^2$) calculated from the equation, were listed in Table S1. The data exhibit good linearities for all the adsorbents with R^2 above 0.998. The

calculating equilibrium adsorption capacity ($q_{e,cal}$) from the pseudo-second-order model were very close to the experimental (q_e).

Table S1 Pseudo-second-order rate equations and constants ofnFe_3O_4@TEPA for fluoride ion (F^-), phosphate or Cr(VI)

anion	equations	k_2 (g/(mg· min))	q_e(mg/g)	$q_{e,cal}$(mg/g)	$k_2\ q_e^2$ (mg/(g· min))	R^2
fluoride	t/q_t= 0.0166 t +0.021	0.013	59.74	60.24	47.61	0.9998
phosphate	t/q_t=0.0195t +0.0174	0.021	50.87	51.28	57.47	0.9998
Cr(VI)	t/q_t=0.0159t + 0.0178	0.014	62.29	62.89	56.18	0.9998

Adsorption kinetic at different temperatures (25 °C~45 °C) were carried out. The Arhenius equation was applied to investigate the E_a for the adsorption process.

$$k = Ae^{-E_a/RT} \qquad (S1)$$

k refers to the pseudo-second-order rate constant ($g\cdot mg^{-1}\cdot min^{-1}$); E_a is the activation energy of anion adsorption ($kJ\cdot mol^{-1}$); A is the pre-exponential factor (frequency factor); R is the gas constant (8.314 $J\cdot mol^{-1}\cdot K^{-1}$); T is the adsorption temperature (K). The results of the linear relationships between ln$k$ and 1/Twere listed in Table S2. With the increasing of temperature, the pseudo-second-order rate constant for all the three kinds of anions increased, which infers that elevated temperature might be favorable a much faster the adsorption rate of nFe_3O_4@TEPAto any of the fluoride ion (F^-), phosphate or Cr(VI)anion. According to the pseudo-second-order rate constant$k$at different temperatures, the activation energies of the adsorption process, E_a,are found to 19.56 kJ/mol , 23.71 kJ/mol , 25.35 kJ/mol for fluoride ion (F^-), phosphate or Cr(VI), respectively. All are less than 42 kJ/mol, which indicate the diffusion process is the rate-controlled step.[2]

Table S2 Pseudo-second-order rate constant at different temperatures (25 °C~45 °C), Arhenius equation and activation energy of anion adsorption

anion	k_2(g/(mg· min))					Arhenius equation	Ea (kJ/mol)
	298	303	308	313	318		
fluoride	0.0102	0.0116	0.013	0.0148	0.0168	lnk=3.3046-2352.3/TR^2= 0.9989	19.56
phosphate	0.016	0.019	0.021	0.026	0.029	lnk=5.4414-2851.3/T R^2= 0.997	23.71
Cr(VI)	0.0098	0.0118	0.014	0.016	0.0188	lnk=5.6147-3048.6/T R^2= 0.9982	25.35

S2. Adsorption capacity of nFe_3O_4@TEPAto anions

The adsorption capacities of nFe_3O_4@TEPA tofluoride ion (F^-), phosphate or Cr(VI) mono-component were investigated by varying the initial concentration of each anion solutions from 50.0 mg/L to 1000 mg/L. Fig. S2 shows that the adsorption capacities of nFe_3O_4@TEPAto any offluoride ion (F^-), phosphate or Cr(VI) increased with the increasing of the initial concentration of the studies anions. The represented parameters using Langmuir and Freundlich adsorption models indicated that for any of the anions,theLangmuirmodels fit better than those of Freundlich isotherms, suggesting a monolayer adsorption.Table S3 presented theLangmuir andFreundlich isotherm parameters as well as the correlation coefficients (R^2) of the adsorption data in the equations.

Table S3 Langmuirand Freundlich isotherm equations and corresponding constants

anion	Langmuir isotherm	Langmuir constants		q_m[b] (mg/L)	Freundlich isotherm	Freundlich constants	
		K_L (L/mg)	$q_{m,c}$ (mg/L)			K_F	$1/n$
fluoride	C_e/q_e = 0.0061 C_e + 0.1513 R^2=0.996	0.04	163.9	163.5	log q_e=0.1345 log C_e+1.8196 R^2= 0.8844	66.0	0.135
phosphate	C_e/q_e = 0.0067C_e + 0.1794 R^2 = 0.998	0.037	149.3	142.5	log q_e=0.2738 log C_e+1.448 R^2 = 0.7117	28.1	0.274
Cr(VI)	C_e/q_e = 0.0025C_e+ 0.0164 R^2= 0.9996	0.152	400	402.4	logq_e=0.2382 log C_e+2.0426 R^2 = 0.663	110.3	0.238

[b]q_m obtained at an initial concentration of 1000.0 mg/L of each ion.

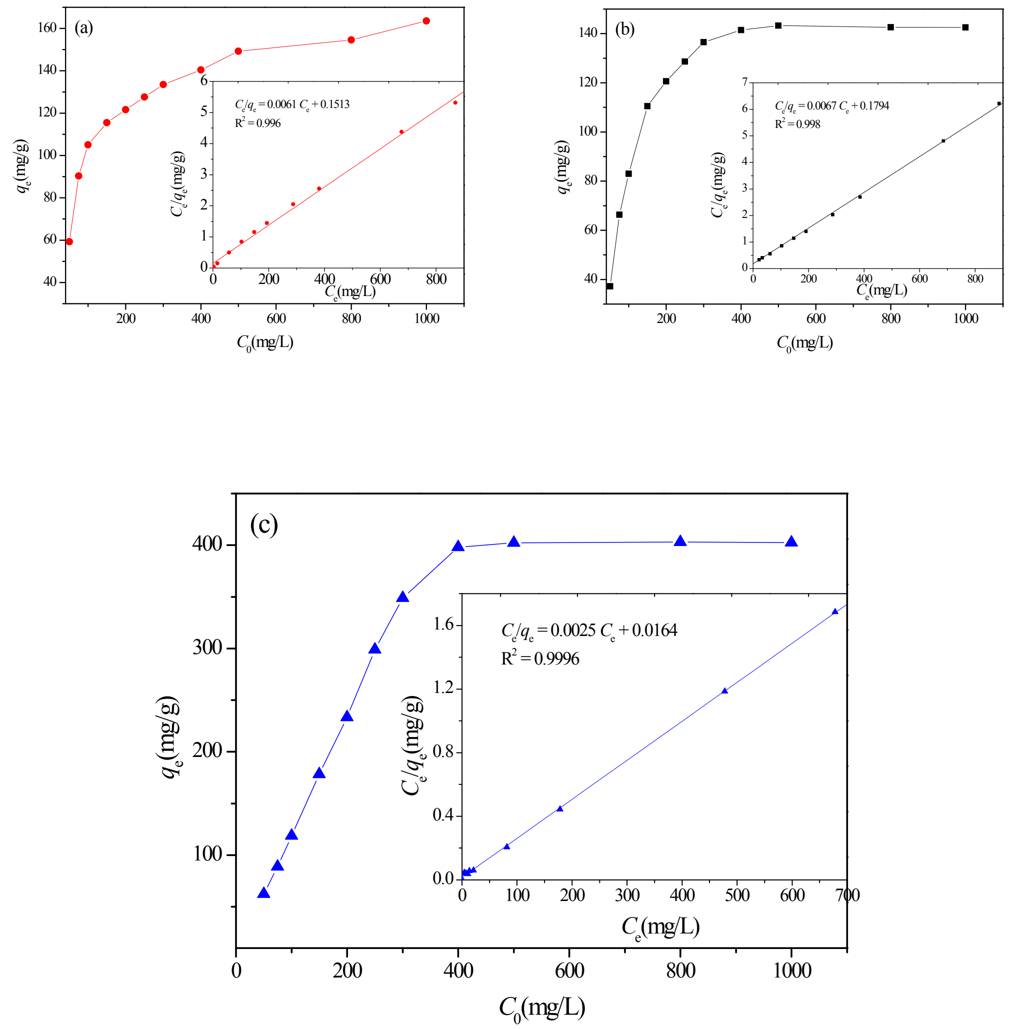

Fig. S2 Adsorption isotherms of mono-componentfluoride ion (F^-), phosphate or Cr(VI) onthenFe_3O_4@TEPA

The thermodynamic parameters,*i.e.*, standard free energy change (ΔG^θ), enthalpy change (ΔH^θ), and entropy change (ΔS^θ), were estimated to evaluate the feasibility and exothermic nature of the adsorption process. The Gibb's free energy change of the process is related to equilibrium constant by eq. (S2):

$$\Delta G^{\theta} = -RT \ln K_D \qquad \text{(S2)}$$

where $K_D$is the distribution coefficient, which is defined as eq. (S3),

$$K_D = \frac{q_e}{C_e} = \frac{\text{Amount of anions adsorbed on Fe}_3\text{O}_4\text{@TEPA}}{\text{Amount of anions in solution}} \times \frac{V}{m} \quad \text{(S3)}$$

According to thermodynamics, the Gibb's free energy change (ΔG^θ) is also

related to the enthalpy change (ΔH^{θ}) and entropy change (ΔS^{θ}) at constant temperature by eq. (S4):

$$\Delta G^{\theta} = \Delta H^{\theta} - T\Delta S^{\theta} \tag{S4}$$

Thus,

$$\ln K_D = \frac{\Delta S^{\theta}}{R} - \frac{\Delta H^{\theta}}{RT} \tag{S5}$$

The values of enthalpy change (ΔH^{θ}) and entropy change (ΔS^{θ}) were calculated from the slope and intercept of the plot of $\ln K_D$ *vs* ($1/T$).The results were given in Table S3. As listed in Table 4, the enthalpy changes (ΔH^{θ}) for the fluoride ion (F^-), phosphate or Cr(VI)were found to be at 38.54, 13.89, 88.03 kJ/mol, respectively, which indicated that the adsorption was endothermic. The entropy changes (ΔS^{θ}) were at 151.2, 50.2, 333.5 J/(mol·K), respectively. The values of ΔG^{θ}were negative for the nFe_3O_4@TEPA to all the studied anions, implying the spontaneous nature of the adsorption process.

Table S4 Thermodynamic parameters for adsorption of nFe_3O_4@TEPA to fluoride ion (F^-), phosphate or Cr(VI) at different temperature (25 ^oC~45 ^oC)

anion	Thermodynamicequation	ΔG (kJ/mol)	ΔH (kJ/mol)	ΔS (J/(mol·K))
fluoride	ln*K* = -4635.2/T+ 18.183<br>R^2 = 0.9976	-6.47~-9.52	38.54	151.2
phosphate	ln*K*= -1671.4/T + 6.0366<br>R^2 = 0.9983	-1.06~-2.05	13.89	50.2
Cr(VI)	ln*K* = -10588/T + 40.231<br>R^2 = 0.9967	-11.6~-18.3	88.03	333.5

S3. Quadratic models for the adsorption capacity of the fluoride ion (F^-), phosphate or Cr(VI), based on BBD ofRSM:

$$q(Cr)=196.35+148.22\times C(Cr(VI))+1.42\times C(F^-)-9.63\times C(P)+5.67\times C(Cr(VI))\times C(F^-) -2.25\times C(Cr(VI))\times C(P)-8.50\times C(F^-)\times C(P)+3.36\times C(Cr(VI))^2-10.79\times C(F^-)^2+1.29\times C(P)^2 \tag{S6}$$

$$q(F)=110.49-0.30\times C(Cr(VI))+39.49\times C(F^{-})-5.31\times C(P)-8.18\times C(Cr(VI))\times C(F^{-})+1.45\times C(Cr(VI))\times C(P)-7.39\times C(F^{-})\times C(P)+0.65\times C(Cr(VI))^{2}-36.22\times C(F^{-})^{2}+0.91\times C(P)^{2} \quad (S7)$$

$$q(P)=80.80-7.16\times C(Cr(VI))-4.40\times C(F^{-})+38.30\times C(P)+6.25\times C(Cr(VI))\times C(F^{-})-8.50\times C(Cr(VI))\times C(P)-4.99\times C(F^{-})\times C(P)+5.53\times C(Cr(VI))^{2}+11.12\times C(F^{-})^{2}-23.27\times C(P)^{2} \quad (S8)$$

S4.Analysis of variance (ANOVA) for the selected quadratic model

Table S5 Process variables and their level for the adsorption ofco-existing ions by BBD

Factors name	Factors code	Level of factors -1	0	1
C_0 Cr(VI) (mg/L)	X_1	10	180	350
C_0 F^- (mg/L)	X_2	10	180	350
C_0 P (mg/L)	X3	10	180	350

Table S6 Experimental design matrix and response

No	Code value X_1	X_2	X_3	Actual value $X_1 C_0$ Cr(VI) (mg/L)	X_2 C_0 F^- (mg/L)	X_3 C_0 P (mg/L)	q Cr(VI) (mg/g)	q F^- (mg/g)	q P (mg/g)
1	0	+1	-1	180	350	10	210.35	132.42	27.29
2	0	-1	+1	180	10	350	180.35	32.71	120.13
3	0	+1	+1	180	350	350	160.35	102.42	100.2
4	+1	0	+1	350	180	350	350.67	112.49	80.2
5	+1	-1	0	350	10	180	320.67	43.77	90.2
6	0	0	0	180	180	180	196.35	110.49	80.8
7	+1	0	-1	350	180	10	360.68	115.6	26.8
8	+1	+1	0	350	350	180	340.68	100.86	95.2
9	-1	-1	0	10	10	180	48.48	32.6	112.2
10	-1	0	+1	10	180	350	45.82	105.6	116.32
11	0	0	0	180	180	180	196.35	110.49	80.8
12	0	0	0	180	180	180	196.35	110.49	80.8
13	0	0	0	180	180	180	196.35	110.49	80.8
14	-1	0	-1	10	180	10	46.82	114.49	28.92
15	0	-1	-1	180	10	10	196.35	33.16	27.32
16	-1	+1	0	10	350	180	45.82	122.42	92.2
17	0	0	0	180	180	180	196.35	110.49	80.8

Table S7ANOVA for Response Surface Quadratic Model (Response1 q(Cr))

Source	Sum of Squares	df	Mean Square	FValue	p-value Prob > F	
Model	1.775E+005	9	19719.45	177.03	< 0.0001	significant
A-C(Cr(VI))	*1.758E+005*	*1*	*1.758E+005*	*1577.81*	*< 0.0001*	*
B-C(F-)	*16.10*	*1*	*16.10*	*0.14*	*0.7151*	
C-C(P)	*741.32*	*1*	*741.32*	*6.66*	*0.0365*	*
AB	*128.48*	*1*	*128.48*	*1.15*	*0.3185*	
AC	*20.30*	*1*	*20.30*	*0.18*	*0.6823*	
BC	*289.00*	*1*	*289.00*	*2.59*	*0.1513*	
A^2	*47.39*	*1*	*47.39*	*0.43*	*0.5350*	
B^2	*490.43*	*1*	*490.43*	*4.40*	*0.0741*	
C^2	*7.03*	*1*	*7.03*	*0.063*	*0.8088*	
Residual	779.73	7	111.39			
Lack of Fit	*779.73*	*3*	*259.91*			
Pure Error	*0.000*	*4*	*0.000*			
Cor Total	1.783E+005	16				

R^2 = 0.9956，Adj R^2 =0.9900，Pred R^2 = 0.9300，Adeq Precision = 40.107
* significant

Table S8 ANOVA for Response Surface Quadratic Model (Response 2 q(F))

Sum of Source	Squares	Mean df	F Square	p-value Value	Prob > F	
Model	18728.99	9	2081.00	100.01	< 0.0001	significant
A-C(Cr(VI))	*0.71*	*1*	*0.71*	*0.034*	*0.8583*	
B-C(F-)	*12472.52*	*1*	*12472.52*	*599.41*	*< 0.0001*	*
C-C(P)	*225.25*	*1*	*225.25*	*10.83*	*0.0133*	*
AB	*267.81*	*1*	*267.81*	*12.87*	*0.0089*	*
AC	*8.35*	*1*	*8.35*	*0.40*	*0.5465*	
BC	*218.30*	*1*	*218.30*	*10.49*	*0.0143*	*
A^2	*1.75*	*1*	*1.75*	*0.084*	*0.7801*	
B^2	*5524.50*	*1*	*5524.50*	*265.50*	*< 0.0001*	*
C^2	*3.49*	*1*	*3.49*	*0.17*	*0.6945*	
Residual	145.65	7	20.81			
Lack of Fit	*145.65*	*3*	*48.55*			
Pure Error	*0.000*	*4*	*0.000*			
Cor Total	18874.65	16				

R^2 = 0.9923，Adj R^2 =0.9824，Pred R^2 = 0.8765，Adeq Precision = 28.530;
* significant

Table S9 ANOVA for Response Surface Quadratic Model (Response 3 q(P))

Source	Sum of Squares	df	Mean Square	F Value	p-value Prob > F	
Model	15636.11	9	1737.35	96.19	< 0.0001	significant
A-C(Cr(VI))	*409.55*	*1*	*409.55*	*22.67*	*0.0021*	*
B-C(F-)	*155.06*	*1*	*155.06*	*8.58*	*0.0220*	*
C-C(P)	*11733.59*	*1*	*11733.59*	*649.63*	*< 0.0001*	*
AB	*156.25*	*1*	*156.25*	*8.65*	*0.0217*	*
AC	*289.00*	*1*	*289.00*	*16.00*	*0.0052*	*
BC	*99.80*	*1*	*99.80*	*5.53*	*0.0510*	
A^2	*128.65*	*1*	*128.65*	*7.12*	*0.0321*	*
B^2	*520.88*	*1*	*520.88*	*28.84*	*0.0010*	*
C^2	*2279.48*	*1*	*2279.48*	*126.20*	*< 0.0001*	*
Residual	126.43	7	18.06			
Lack of Fit	*126.43*	*3*	*42.14*			
Pure Error	*0.000*	*4*	*0.000*			
Cor Total	15762.54	16				

R^2 = 0.9920， Adj R^2 =0.9817， Pred R^2 = 0.8717， Adeq Precision = 28.714

* significant

Table S10 The relative percentages of the main elements relatively weight (w%) of nFe_3O_4@TEPA before and afteradsorption

Elements relatively weight (w%)	before adsorption	afteradsorption
Fe 2p	29.28	28.62
O 1s	26.02	25.12
N 1s	17.52	15.65
C 1s	27.23	27.03
Cr 2P	0	1.88
P 2p	0	1.01
F 1s	0	0.78

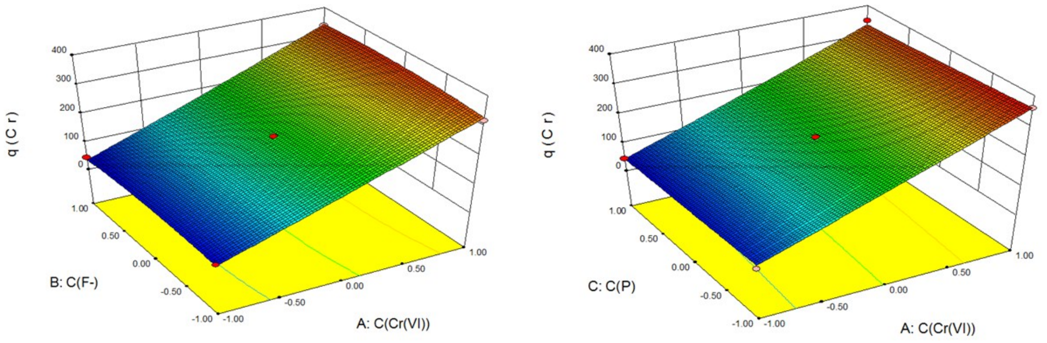

(a) (b)

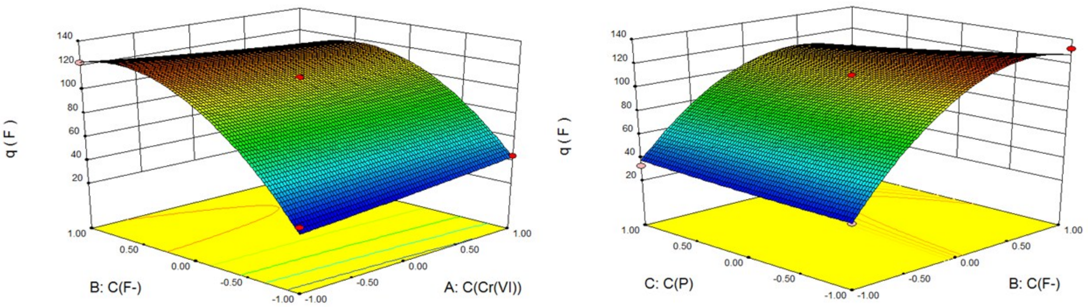

(c) (d)

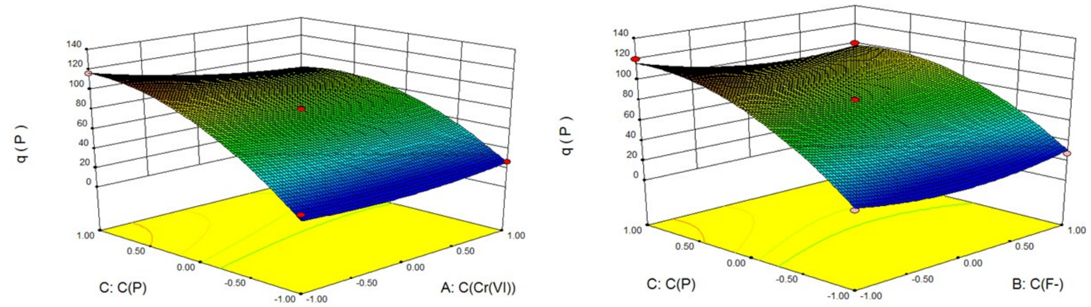

(e) (f)

Fig. S3 The effect of the co-existing fluoride ion to the adsorption of Cr(VI) (a),the effect of the co-existing phosphate to the adsorption of Cr(VI) (b), the effect of the co-existing Cr(VI)to the adsorption of fluoride ion (c),the effect of the co-existing phosphateto the adsorption of fluoride ion (d),the effect of the co-existing Cr(VI)to the adsorption of phosphate (e),the effect of the co-existing fluoride ionto the adsorption of phosphate (f)

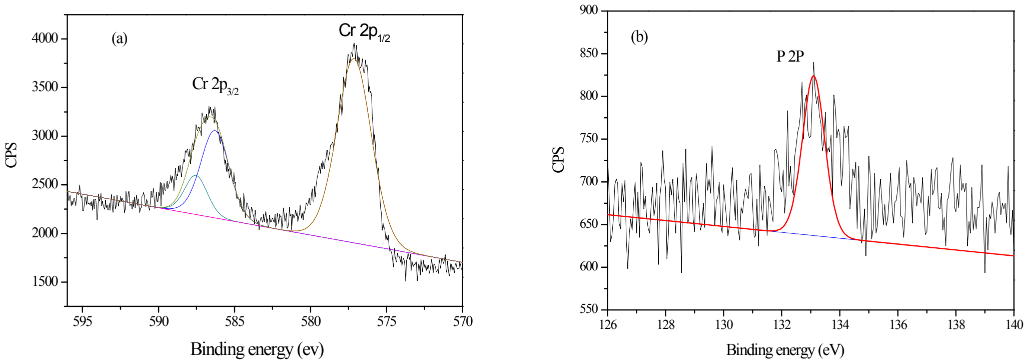

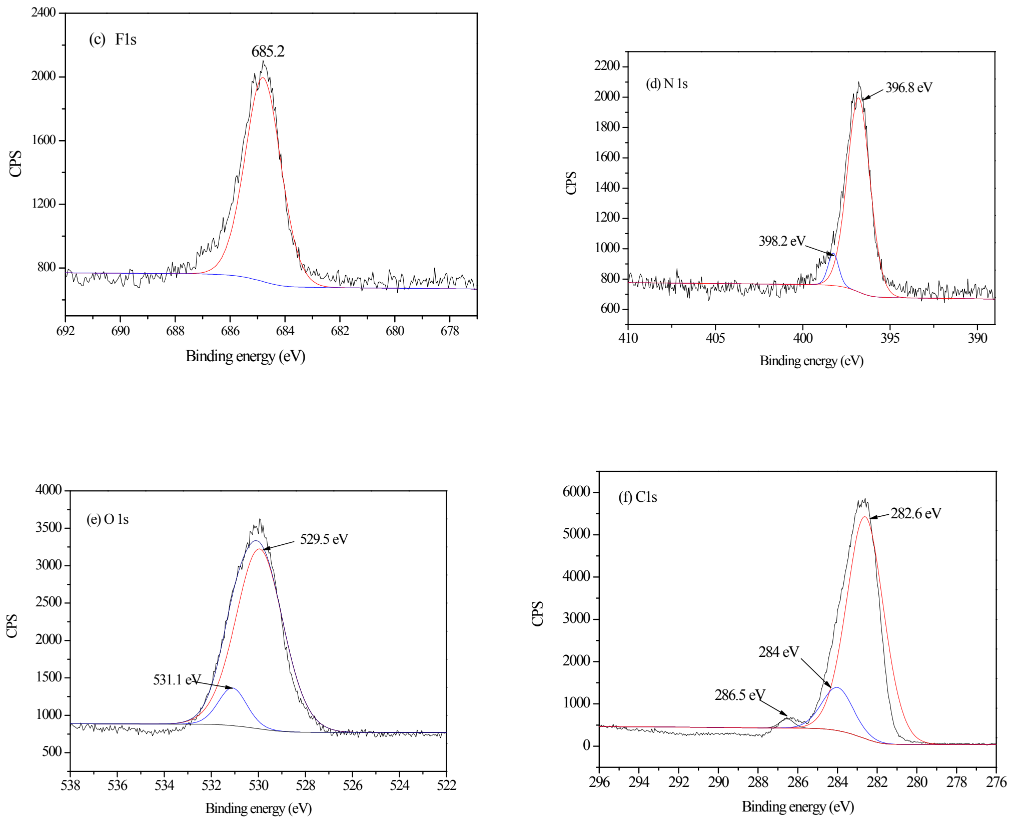

Fig.S4 High-resolution XPS spectra of nFe_3O_4@TEPA after adsorption of the co-existing the three anions (a) Cr2P; (b)P1p; (c) F1s; (d) N1s; (e) O1; (f) C1s.
